# Renal Carcinoma Patterns and Prevalence in Bahrain: A Descriptive Study

**DOI:** 10.7759/cureus.31443

**Published:** 2022-11-13

**Authors:** Ali Al Aradi, Ahmed A Al Rashed, Mohamed Mubarak, Omran Hasan, Ameer Al Arayedh, Qasim M Isa, Husain Alaradi

**Affiliations:** 1 Urology, Salmaniya Medical Complex, Manama, BHR; 2 Surgery, Salmaniya Medical Complex, Manama, BHR

**Keywords:** urological cancer, urology and oncology, renal neoplasm, kidney pathology, kidney disease

## Abstract

Cancer is a major health problem with a significant impact on society and healthcare systems. In 2018, approximately 18.1 million cases of cancer were diagnosed and 9.6 million deaths were documented. Urological cancers account for 12.9% of new cases recorded and 8% of deaths due to cancer worldwide. The latest cancer registries covering the Gulf Cooperation Council (GCC) countries report that 4078 cases of renal cell carcinoma were diagnosed from 1998 to 2012. Urological cancers comprised 9.4% of all cases with an incidence rate of 16.1% in males and 3.2% in females.

All renal cancer cases documented in Salmaniya Medical Complex (SMC) from 2014 to 2018 were reviewed. Data collected for all patients from the electronic health record system included age at diagnosis, gender, laterality of cancer (where applicable), histological type, and TNM (tumor, node, metastasis) classification and staging. Furthermore, World Health Organization (WHO) grade and data were collected for kidney cancer cases. Statistical analysis was carried out using Statistical Package for the Social Sciences (SPSS) version 23 (IBM Corp, Armonk, NY).

From 2014 to 2018, there were 65 documented cases of kidney cancer with an average caseload of 13 cases per year. The mean age at diagnosis was 57.6 years. Clear cell carcinoma was the most common histological subtype (37.5%). Stage 1 was the most common stage at diagnosis (35.4%) and the age-standardized mortality rate for males and females were 4.59 and 4.58 in 100,000, respectively.

Kidney cancer is a urological malignancy that can pose a burden on both the patient and the healthcare system. There should be a national effort to better understand the etiology and epidemiology of this disease entity with regard to our population. Such efforts would make data regarding diagnosis, management, and follow-up more accessible and would add positively to our healthcare system.

## Introduction

Kidney cancer is a common urological malignancy with renal cell carcinoma (RCC) being the most encountered variant of renal cancers [1]. Around 18 million new cases of cancer are diagnosed annually, wherein RCC makes up about 2.2% of the overall incidence [1]. Furthermore, it contributes to around 1.8% of cancer mortalities per annum and Siegel et al reported that it contributes to 5% and 3% of all oncological diagnoses in males and females, respectively [2]. It is generally accepted that it is a disease of old age with a typical age at presentation ranging from 50 to 70 years. However, with recent advances in diagnostic imaging, it is not unusual to diagnose at younger ages [2,3].

The main histological subtypes of renal cancer include clear cell, papillary, and chromophobe carcinoma [4]. Most cases are sporadic with only 2-3% associated with familial inheritance per GLOBOCAN reports [5]. Znaor et al. report that incidence rates vary 15-fold worldwide, with higher rates reported in North America and Europe in comparison to lower rates in South-East Asia and Africa [6]. The latest cancer registries covering the Gulf Cooperation Council (GCC) countries report that 4078 cases of RCC were diagnosed from 1998 to 2012 with an age-standardized mortality rate (ASR) of 3.1 per 100,000 in males and 2 per 100,000 in females [7]. The same report also shows that a total of 4758 cancer cases were diagnosed in Bahrain within the same period. RCC constituted around 2% of these cases and had an ASR of 5.1 and 2.7 per 100,000 in males and females, respectively [7].

RCC carries a heavy financial and logistical burden on the healthcare system due to the nature of its diagnostic workup, which requires different laboratory and imaging modalities suited to the patient, costly management options that include both surgical and medical approaches, and regular follow-up [7]. All these factors create a need for improved awareness campaigns, efficient workup, and management guidelines. Despite the magnitude of the disease, there is a paucity of information about RCC on a local scale. In this study, we aimed to evaluate the patterns of kidney cancer cases in Salmaniya Medical Complex (SMC), Kingdom of Bahrain from 2014 to 2018.

## Materials and methods

SMC is the main public hospital in the Kingdom of Bahrain. It is a tertiary center, which covers the majority of the population's healthcare needs in the country. The data were collected using the electronic healthcare information system (ISEHA). This data included the age at diagnosis, the patient’s gender, laterality of cancer (where applicable), histological type, TNM classification, and finally staging. 

The study was retrospective in its design and all cases of kidney cancer between the years 2014 and 2018 in SMC were recorded and reviewed. Cases from the year 2019 onwards were not recorded due to discrepancies caused by the COVID pandemic, in which newly diagnosed cases were either lost in follow-up or had delayed presentations due to lack of physical attendance and hence did not complete full workups.

The histological types of cancer were collected from either the electronic system or from the pathology department files for those patients in which the reports were not present on the electronic system. However, some patients did not have an electronic or physical histology report and were marked as “missing” in the results section and data analysis.

All statistical analyses were carried out using Statistical Package for the Social Sciences (SPSS) version 23 (IBM Corp, Armonk, NY). The ASR, which is a weighted average of the age-specific mortality rates per 100,000 people, was derived by dividing the deaths in the age group by the estimated population of that age group and then multiplying by 100,000. It aids in determining the mortality risks in the study population compared to the standard population within the same age group.

## Results

**Figure 1:**
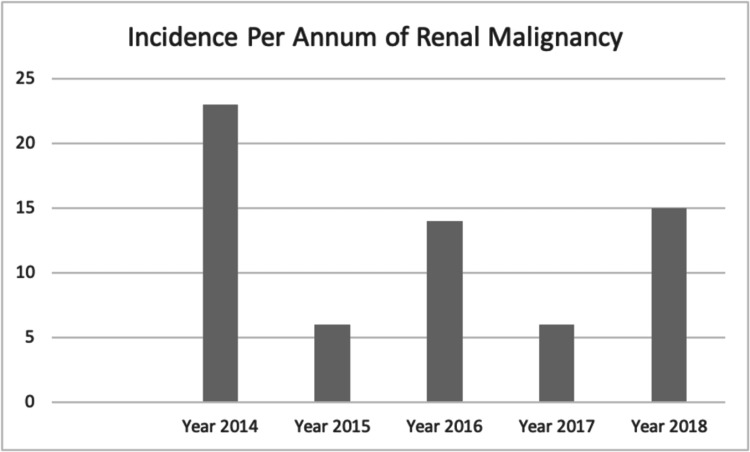
Number of New Cases of RCC Encountered Per Year in SMC RCC, renal cell carcinoma; SMC, Salmaniya Medical Complex.

During 2014-2018, there were a total of 65 documented cases of kidney cancer with an average caseload of 13 cases per year as noted in Figure 1. 

**Figure 2:**
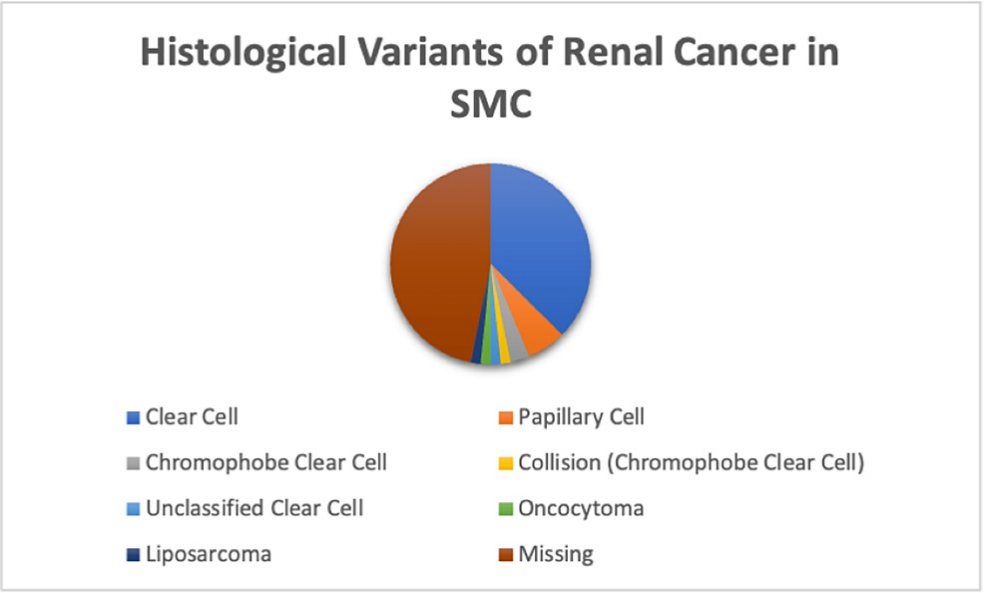
Histological Variants of Renal Cancers within the Total Sample SMC, Salmaniya Medical Complex.

The mean age at diagnosis was 57.6 years with 63% (43 patients) being male in gender. Clear cell carcinoma was the most common histological subtype (37.5%), followed by other carcinoma variants as demonstrated in Figure 2. 

Furthermore, the laterality of the tumor was almost equal being right kidney (37.5%), left kidney (31.5%), and missing (31.5%). The calculated ASR kidney cancer was (4.59/100,000) in males and (4.58/100,000) in females. Finally, Stage 1 was the most common stage at diagnosis (35.4%) with half the sample not having metastasis upon diagnosis. Unfortunately, 39.1% of patients in the sample have missing staging owing to the lack of histological reports which can be seen in Table 1.

**Table 1:** Staging of all Renal Cancers Encountered

TNM Staging		Number of Patients (n)
T		
	T1	21 (32.8%)
	T2	5 (7.8%)
	T3	8 (12.5%)
	T4	2 (3.1%)
	Missing	28 (43.8%)
N		
	N0	34 (53.1%)
	N1	9 (14.1%)
	Missing	21 (32.8%)
M		
	M0	33 (51.6%)
	M1	8 (12.5%)
	Missing	23 (35.9%)
Stage		
	Stage 1	23 (35.9%)
	Stage 2	3 (4.7%)
	Stage 3	7 (10.9%)
	Stage 4	6 (9.4%)
	Missing	25 (39.1%)

## Discussion

Kidney cancer is a common disease and has multiple implications, especially when it comes to the burden it poses on both the patient and the healthcare system. As far as we know, no local studies were previously conducted to investigate the pattern and trends of kidney cancer in Bahrain and therefore the disease has not been completely explored yet in relation to the Bahraini population. 

With the increasing use of improved, accessible imaging modalities, more cases have been diagnosed, thus increasing both the incidence and prevalence. Kidney cancer accounts for 3.2% and 0.9% of Bahraini males and females, respectively, as well as being the third most common urological cancer in Bahrain according to the GCC registry [7]. Most of the cases were detected in the early stages, thus having curative management. ASR for kidney cancer was 4.59/100,000 in males and 4.58/100,000 in females, thus making it lower than the global but higher than the cumulative GCC ASR [7]. To further demonstrate, our ASR for males is higher than that of Saudi Arabia, Oman, and the United Arab Emirates (2.5, 2.3, and 2 per 100,000) but is lower than that of Kuwait and Qatar (5.1 and 5.6 per 100,000) [7].

On the other hand, we had a higher ASR for females than the other five GCC states (Saudi Arabia: 1.7, Oman: 1.9, Qatar: 3.4, Kuwait: 2, and United Arab Emirates: 1.1 per 100,000) [7]. ASR for kidney cancer in Europe, the United States, Korea, Hong Kong, and Taiwan were all higher than that of our population (4.1-31.4 males and 2.1-14.5 females, 17, 5.9, 4.8, 5.32 per 100,000) [8,9]. However, the ASR in China was notably lower (3.35 per 100,000). The causes of possibly lower ASR rates could be due to lower consumption of tobacco products and body mass index (BMI) levels [10].

The clinical presentation of kidney cancer is extremely variable on a case-to-case basis. Many cases may go asymptomatic until discovered incidentally on imaging done for other purposes. In fact, Silverman et al. report that more than 60% of RCCs are detected incidentally [11]. Symptoms range from flank pain, hematuria, and abdominal mass, which indicate a localized disease to systemic syndromes such as hypertension, anemia, and cachexia [11]. Furthermore, kidney cancer is notoriously associated with lifestyle factors that increase the risk of incidence. Smoking is the most commonly associated risk factor with multiple studies having already established the relationship between smoking and increased risk for kidney cancer. A hazard ratio of 1.58 (95% CI 1.09-2.29) was established by the VITAL study, with a similar result being reported in another trial evaluating the association of smoking intensity with the risk of developing RCC [12].

Furthermore, a meta-analysis of more than 24 papers reported a relative risk of incidence of 1.31 (1.22-1.4), 1.36 (1.19-1.56), and 1.16 (1.08-1.25) for all smokers, current smokers, and former smokers, respectively [13]. Additionally, BMI has also been implicated as a big risk factor for kidney cancer. High-fat diets for prolonged periods have been shown to be associated with cancer risk [14]. Moreover, a prospective cohort study confirmed that obesity is associated with RCC, whereas a BMI >35 has a hazard ratio of 1.71 (95% CI 1.06-2.79) [15]. Other studies suggest that smoking increases the risk of RCC by 50% in males and 20% in females, while 5 kg/m^2^ increases in BMI increase the risk of RCC by 24% in males and 34% in females [15].

Clinical examination plays a minor role in the workup of kidney cancer, with red flags such as palpable abdominal mass, lymph nodes, and irreducible varicoceles prompting an immediate radiological investigation. Although both ultrasound and computed tomography (CT) scans may be used for diagnosis, in our center we prefer the use of a CT scan of the abdomen and pelvis. Magnetic resonance imaging (MRI) may also be used in a select of patients who are not fit for a CT scan or may have venous involvement. In our institution, we offer surgical options of partial or complete nephrectomy based on the suitability of the candidate. For more advanced stages, immunotherapy and targeted therapy are integrated as the mainstay of treatment with both treatment modalities being carried out by the oncology department. Furthermore, other modalities such as cryotherapy and thermoablation therapy are not offered at our center. 

Unfortunately, we do not have sufficient follow-up and mortality data to help us further evaluate and assess the status of kidney cancer in Bahrain. This poses a clear limitation to our study. We believe that the availability of such data would help us tackle this disease in a much better and holistic manner. Moreover, missing data made it difficult to fully evaluate kidney cancer in our institution and be on par with other studies reporting on the same topic.

## Conclusions

Kidney cancer is a common urological malignancy that can pose a heavy burden on both the patient and the healthcare system. We documented a total of 65 cases over five years and an ASR of 4.59 and 4.58 in 100,000 in males and females, respectively. Although screening programs are not common due to the low incidence rates of this malignancy, we believe that there should be a national effort to better understand the etiology and epidemiology of this disease entity with regard to our population. Such efforts would make data regarding the prevention, diagnosis, management, and follow-up reachable, controllable, and would add positively to our healthcare system.
